# Bootstrap calibration of TRANSMIT for informative missingness of parental genotype data

**DOI:** 10.1186/1471-2156-4-S1-S39

**Published:** 2003-12-31

**Authors:** Andrew S Allen, Julianne S Collins, Paul J Rathouz, Craig L Selander, Glen A Satten

**Affiliations:** 1 Department of Biostatistics and Bioinformatics and Duke Clinical Research Institute, Duke University, Durham, North Carolina, USA; 2 JC Self Research Institute, Greenwood Genetic Center, Greenwood, South Carolina, USA; 3 Department of Health Studies, University of Chicago, Chicago, Illinois, USA; 4 National Center for Environmental Health, Centers for Disease Control and Prevention, Atlanta, Georgia, USA

## Abstract

Informative missingness of parental genotype data occurs when the genotype of a parent influences the probability of the parent's genotype data being observed. Informative missingness can occur in a number of plausible ways and can affect both the validity and power of procedures that assume the data are missing at random (MAR). We propose a bootstrap calibration of MAR procedures to account for informative missingness and apply our methodology to refine the approach implemented in the TRANSMIT program. We illustrate this approach by applying it to data on hypertensive probands and their parents who participated in the Framingham Heart Study.

## Background

Missing parental genotype data is a common problem in association studies utilizing parental controls and has led to the development of a variety of approaches aimed at extending standard methods such as the transmission-disequilibrium test (TDT) to allow for a parent's genotype to be missing [1-4]. A common assumption made in such approaches [1,2] is that parental missingness is not related to the underlying, unobserved, genotype of the missing parent. This assumption is referred to as noninformative missingness or missingness at random (MAR) and allows reconstruction of the missing parent's genotype using the genotype frequencies among the observed parents and constraints imposed by spouse and offspring genotypes. Informative missingness, on the other hand, occurs when a parent's missingness is related to his or her genotype at the locus of interest. In this case, the distribution of genotypes in missing parents cannot be immediately constructed from the distribution of genotypes among parents in intact trios. Therefore, when informative missingness is present, procedures that assume MAR will tend to reconstruct the genotypes of missing parents incorrectly, leading to biased results.

Informative missingness can occur in several ways. First, the parent's genotype may in fact be associated with the disease of interest which in turn, if manifest in the parent, may cause or influence missingness. Second, the genotype may be associated with a different disease that results in parental missingness. Finally, informative missingness can arise strictly as an artifact of population stratification. Consider a population comprising a number of subpopulations with varying allele frequencies and assume that the probability of a parent being missing also varies across these subpopulations. Even though there may be no relationship between missingness and parental genotype in any given subpopulation, in the overall population, the two may be correlated, resulting in informative missingness. Note that the second and third situations described above affect the null distribution of parental controlled association tests and can, as a result, lead to invalid inference. The first situation only affects the alternative hypothesis and hence only affects power.

Allen et al. [5] developed parental controlled association tests that are valid when parental genotype data are informatively missing. This approach retains comparable power when the data are MAR but assumes the marker being considered is bi-allelic. Here we propose a new approach to multiallele association testing that is robust to informative missingness. This approach uses a multiallele extension of the missingness model presented in Allen et al. [5] and a bootstrapping procedure to recalibrate MAR-based methods to account for informative missingness. In the next section we present the missingness model and how the bootstrap calibration procedure can be used to correct MAR-based TRANSMIT results for informative missingness. We illustrate our methodology by applying it, as well as an unadjusted MAR-based approach, to data on hypertensive probands and their parents who participated in the Framingham Heart Study. We conclude with a discussion of the results of this analysis including the possibility of informative missingness in this data set.

## Methods

### A model for informative missingness

Assume a trio-based sampling design in which *N *individuals with disease or trait of interest (denoted by *D *= 1) are sampled and, when possible, their parents are also recruited. At a locus of interest, let the proband genotype be denoted *G*_*o *_and let *G*_*f *_(*G*_*m*_) denote the paternal (maternal) genotypes. Let *G*_*p *_= (*G*_*f*_, *G*_*m*_) and assume there are *K *alleles at the locus of interest. Let *R *be an indicator of missingness so that if neither parent is missing, *R *= (0, 1) if the father but not the mother is missing, *R *= (1, 0) if the mother but not the father is missing, and *R *= (0, 0) if both parents are missing. Let  and  denote the observed and missing parental genotype information respectively, so that for *R *= (1, 0)  = *G*_*f *_and  = *G*_*m*_. If *R *= (1, 1), then  is the empty set and  = *G*_*p*_. If we define  and  then Allen et al. [5] show that the conditional likelihood of *G*_*o*_,  given missingness and the offspring being diseased can be written as



where *Pr*(*G*_*o*_|*G*_*p*_, *D *= 1)are the transmission probabilities conditional on parental genotype given by Schaid and Sommer [6]. Over the entire sample, the likelihood is



where *G*_*oi *_and  are the offspring genotype and observed parental information for the *i*^th ^proband.

In order to specify equation (1) fully we need a model for θ_*R *_(*G*_*p*_) and *p*_0 _(*G*_*p*_) (we will only be optimizing this likelihood under the null so *Pr*(*G*_*o*_|*G*_*p*_, *D *= 1) is purely combinatoric). We assumed assortative mating and allowed for departures from Hardy-Weinberg equilibrium by estimating the value of fixation index *F *[7]. Specifically, we assume *p*_0 _(*G*_*p*_) = *p*_0 _(*G*_*f*_) *p*_0 _(*G*_*m*_) where



and where genotype *G *consists of alleles *k *and *l *with allele frequencies π_*k *_and π_*l*_, respectively. We arrived at a log-linear model forθ_*R*_(*G*_*p*_). Specifically, we take



where  are the number of copies of allele *k *in the father's (mother's) genotype. This model was found to be both well identified and rich enough to handle a variety of realistic missing data scenariosallowing for differential maternal () and paternal () effects on an individual's missingness as well as an effect due to his or her spouse ().

### Bootstrap calibration of MAR procedures

As mentioned above, tests derived from procedures assuming MAR will have improper size when informative missingness holds. In particular, our simulations show that Clayton's approach as implemented in the TRANSMIT program [2] can result in greatly inflated type I error rates when presented with plausible informative missingness scenarios. We propose a bootstrap calibration of MAR-based tests using the informative missingness model presented above. The procedure, applied to the TRANSMIT approach of Clayton [2], is as follows.

First, we fit the informative missingness model by maximizing the conditional likelihood (equation (1)) under the null hypothesis of no transmission disequilibrium to obtain estimates . Note that under the null, *Pr*(*G*_*o*_|*G*_*p*_, *D *= 1) is made up of known constants and need not be estimated.

With these parameters, we performed the following procedure:

1. Sampled parental data from Pr(*G*_*m*_, *G*_*f*_|*D *= 1, *R*; ).

2. Given the full set of parental data, we imputed offspring data given the parental data by randomly sampling an allele from each parent's genotype.

3. Calculated the test statistic *T *testing the null hypothesis of no association between disease and marker (or a particular allele) via TRANSMIT, using the imputed offspring data and the sampled parental data with the originally unobserved parent (if any) set to missing.

Steps 1–3 were repeated until *B *replicates had been obtained (we used *B *= 999). The 100 × (1 - α)^th ^percentile of the empirical distribution of the test statistics {*T*_1_,...,*T*_*B*_} was taken as the critical value of an α-level test calibrated for informative missingness. A test statistic *t *obtained from TRANSMIT applied to the original data can then be compared with this critical value to determine the significance of the results.

### Example data

We applied this approach to 224 nuclear families extracted from the Framingham Heart Study pedigree data provided by Genetic Analysis Workshop 13 (GAW13). Individuals were selected based on the presence of both phenotypic and genotypic information. Individuals were given a hypertensive phenotype if they had hypertension at any exam or were taking medication for hypertension. Of nuclear families with at least one affected offspring, 77 had complete parental data; 96 had only maternal data; and 51 had only paternal data. We excluded probands without any parental genotype data because they are likely to contribute little information. For families with more than one affected offspring we randomly selected one proband. We focused on the chromosome 17q21-q23 region, which had been linked to hypertension (as a quantitative trait) in previous studies [8,9], containing markers GATA25A04 and ATC6A06 [8] as well as GATA49C09 [9]. Rare alleles were pooled with nearest repeats to maintain stable estimates. We tested for association between alleles at these markers and the hypertension phenotype using both the bootstrap recalibration procedure (adjusted for informative missingness) and the unadjusted (i.e., without the bootstrapping procedure) MAR-based results from TRANSMIT. The results of our analysis are presented in Table 1.

## Results

The bootstrap-calibrated and MAR-based inferences corresponded well on marker GATA25A04. Results on markers GATA49C09 and ATC6A06 showed more discrepancies. Quantitative differences were evident at many of the alleles on these markers, especially the combined alleles 158 and 166 of marker GATA49C09. Though these effects for any given allele were marginal for ATC6A06, differences between the two procedures' overall chi-square tests at each marker were more substantial. An analysis of intact trios supported the conclusions of the bootstrap-calibrated inferences, finding no association with combined alleles 158 and 166 of marker GATA49C09 (results not shown). The intact trio analysis is valid under certain types of informative missingness, though at a loss of power relative to our bootstrap calibration approach.

## Discussion

The discrepancies seen in this analysis between the MAR-based and the bootstrap-calibrated tests may be due to the presence of informative missingness at markers GATA49C09 and ATC6A06 in this data set. This conclusion is supported by the intact trio analysis. In addition, the differences between the MAR-based and bootstrap-calibrated *p*-values observed were consistent with those documented in simulations [5]. In these simulations, informative missingness causes MAR-based procedures to yield smaller-than-warranted *p*-values, leading to an increased type I error rate. Moreover, informative missingness is certainly plausible in this region due to its close proximity to a number of cancer genes, including *BRCA1*. Further data including a denser marker set in this region will be helpful in confirming this possibility.

On the surface, it may appear that the lack of parental genotype information would make the problem of informative missingness intractable, or worse, that modelling informative missingness could lead to biased results through the introduction of unverifiable assumptions. However, there is, in fact, sufficient information in the way of constraints imposed by spouse and offspring genotypes to make estimation of the effect of genotype on missingness not only tractable but more robust than the standard MAR-based analysis. Simulations suggest that even very mild informative missingness can have an enormous impact on the size of MAR-based tests [5]. The bootstrap calibration approach proposed here protects against this inflation with minimal impact on power.

## Figures and Tables

**Table 1:** Comparison of boostrap-calibrated and MAR-based tests

**Marker**	**Allele(s)**	**MAR Chi Square (df)**	**MAR *P*-value**	**Bootstrap *P*-value**
GATA25A04		1.5966 (5)	0.902	0.938
	184 & 188	0.003 (1)	0.956	0.955
	192	0.334 (1)	0.564	0.600
	196	0.506 (1)	0.477	0.481
	200	0.173 (1)	0.677	0.778
	204	0.111 (1)	0.739	0.754
	208	0.685 (1)	0.408	0.426
ATC6A06		9.325 (5)	0.097	0.378
	113	1.111 (1)	0.292	0.395
	116	1.212 (1)	0.271	0.328
	119	0.411 (1)	0.522	0.784
	122	1.356 (1)	0.244	0.576
	125	4.162 (1)	0.041	0.047
	128 & 131	1.482 (1)	0.223	0.358
GATA49C09		10.472 (10)	0.400	0.722
	158 & 166	4.062 (1)	0.044	0.176
	170	0.053 (1)	0.819	0.828
	174	1.25 × 10^-6 ^(1)	0.999	0.999
	178	0.012 (1)	0.914	0.919
	182	0.015 (1)	0.902	0.949
	186	0.809 (1)	0.368	0.393
	190	0.193 (1)	0.661	0.751
	194	0.034 (1)	0.855	0.921
	198	1.758 (1)	0.185	0.306
	202	3.838 (1)	0.050	0.059
	206 & 210 & 214	0.201 (1)	0.654	0.749

## References

[B1] Weinberg CR (1999). Allowing for missing parents in genetic studies of case-parent triads. Am J Hum Genet.

[B2] Clayton DA (1999). Generalization of the transmission/disequilibrium test for uncertain haplotype transmission. Am J Hum Genet.

[B3] Sun FZ, Flanders WD, Yang QH, Khoury MJ (1998). A new method for estimating the risk ratio in studies using case-parental control design. Am J Epidemiol.

[B4] Sun FZ, Flanders WD, Yang QH, Khoury MJ (1999). Transmission disequilibrium test (TDT) when only one parent is available: the 1-TDT. Am J Epidemiol.

[B5] Allen AS, Rathouz PJ, Satten GA (2003). Informative missingness in genetic association studies: case-parent designs. Am J Hum Genet.

[B6] Schaid DJ, Sommer SS (1993). Genotype relative risks-methods for design and analysis of candidate-gene association studies. Am J Hum Genet.

[B7] Hartl DL, Clark AG (1997). Principles of Population Genetics. Sunderland, MA, Sinauer Associates.

[B8] Levy D, DeStefano AL, Larson MG, O'Donnell CJ, Lifton RP, Gavras H, Cupples A, Myers RH (2000). Evidence for a gene influencing blood pressure on chromosome 17. Hypertension.

[B9] O'Donnell CJ, Lindpainter K, Larson MG, Rao VS, Ordovas JM, Schaefer EJ, Myers RH, Levy D (1998). Evidence for association and genetic linkage of the angiotensin-converting enzyme locus with hypertension and blood pressure in men but not women in the Framingham Heart Study. Circulation.

